# Effects of Rho1, a small GTPase on the production of recombinant glycoproteins in *Saccharomyces cerevisiae*

**DOI:** 10.1186/s12934-016-0575-7

**Published:** 2016-10-21

**Authors:** Sha Xu, Ge-Yuan Zhang, Huijie Zhang, Toshihiko Kitajima, Hideki Nakanishi, Xiao-Dong Gao

**Affiliations:** 1 School of Biotechnology, Key Laboratory of Glycobiology and Biotechnology, Ministry of Education, Jiangnan University, 1800 Lihu Road, Wuxi, 214122 Jiangsu China; 2 State Key Laboratory of Food Science and Technology, Jiangnan University, Wuxi, 214122 Jiangsu China

**Keywords:** Humanized *N*-glycosylation, Recombinant-protein production, Rho1p GTPase, Cell wall integrity, *Saccharomyces cerevisiae*

## Abstract

**Background:**

To humanize yeast *N*-glycosylation pathways, genes involved in yeast specific hyper-mannosylation must be disrupted followed by the introduction of genes catalyzing the synthesis, transport, and addition of human sugars. However, deletion of these genes, for instance, *OCH1,* which initiates hyper-mannosylation, could cause severe defects in cell growth, morphogenesis and response to environmental challenges.

**Results:**

In this study, overexpression of *RHO1*, which encodes the Rho1p small GTPase, is confirmed to partially recover the growth defect of *Saccharomyces cerevisiae* Δ*alg3*Δ*och1* double mutant strain. In addition, transmission electron micrographs indicated that the cell wall structure of *RHO1*-expressed cells have an enhanced glucan layer and also a recovered mannoprotein layer, revealing the effect of Rho1p GTPase on cell wall biosynthesis. Similar complementation phenotypes have been confirmed by overexpression of the gene that encodes Fks2 protein, a catalytic subunit of a 1,3-β-glucan synthase. Besides the recovery of cell wall structure, the *RHO1*-overexpressed Δ*alg3*Δ*och1* strain also showed improved abilities in temperature tolerance, osmotic potential and drug sensitivity, which were not observed in the Δ*alg3*Δ*och1*-*FKS2* cells. Moreover, *RHO1* overexpression could also increase *N*-glycan site occupancy and the amount of secreted glycoproteins.

**Conclusions:**

Overexpression of *RHO1* in ‘humanized’ glycoprotein producing yeasts could significantly facilitate its future industrial applications for the production of therapeutic glycoproteins.

**Electronic supplementary material:**

The online version of this article (doi:10.1186/s12934-016-0575-7) contains supplementary material, which is available to authorized users.

## Background

Therapeutic proteins have been widely used in inflammatory diseases, cancer, neurological disorders and diabetes [1]. A majority of therapeutic proteins display one or more post-translational modifications, which invariably influence the biochemical and therapeutic properties of these proteins. About 70 % of therapeutic proteins are glycoproteins [2] and their glycan parts have numerous structural, functional and regulatory roles [3]. Glycosylation can influence a variety of physiological processes, including intracellular targeting, protein–protein binding and molecular stability [4]. There are five types of glycosylation, *N*-, *O*-, *P*-, *C*-, and *G*-linked [5]. All of them involve the addition of an oligosaccharide structure to the protein core but through different binding sites. *N*-Glycosylation is one of the most prevalent but structurally most complex chemical modifications that occurs naturally in proteins.

Most therapeutic glycoproteins are currently produced by mammalian cells. Chinese hamster ovary (CHO) cells are the most commonly used higher eukaryotic cells for the production of glycoproteins [6]. However, due to limited growth rate, expensive serum-based media, and the potential spread of infectious diseases, production of glycoproteins in CHO cells results in low productivity and an economically harsh process. Relative to mammalian cell culture, yeasts offer generally high yields of recombinant proteins, are well-characterized, give serum-free growth media, are readily adapted to large-scale fermentation processes and have greatly reduced costs [7]. Humanized glycoproteins have already been produced in a variety of yeasts, such as *Saccharomyces cerevisiae* [8], *Pichia pastoris* [9, 10], *Yarrowia lipolytica* [11], *Hansenula polymorpha* [12], *Schizosaccharomyces pombe* [13] and *Ogataea minuta* [14, 15].

Although human and yeast cells share the initial stages of the *N*-glycosylation pathway in the endoplasmic reticulum (ER), further modification in the Golgi apparatus is highly different. The final glyco-form of human *N*-glycans are mainly of the complex or hybrid type [7]. In contrast, yeast cells provide a yeast specific hypermannosylated type *N*-glycosylation on recombinant glycoproteins, which are antigenic in humans. If these are used for the therapeutic purpose, such glycoproteins will be cleared rapidly from the bloodstream due to interaction with human mannose receptors, and could also cause immunogenic reactions in humans [16]. To mimic the human type of glycosylation, yeast cells have been glyco-engineered by disruption of genes encoding specific mannosyltransferases (e.g. *ALG3* and *OCH1*) causing a loss of hyper-mannosylated structures followed by the expression of exogenous genes catalyzing the synthesis, transport, and modification of human sugars [16]. However, the Δ*alg3* mutation causes under-occupancy of *N*-glycosylation sites [17]. Moreover, deletion of genes that encode specific glycosyltransferases, like *OCH1*, causes hypo-glycosylation of yeast cell proteins that leads to a direct rearrangement of cell wall constituents [18]. Fungal cell wall integrity plays an important role in cell growth and survival from various environmental stresses [19], thus its damage reveals a severe growth defect and a decrease in protein production [20, 21]. During the past decade, considerable efforts have been made to overcome this serious drawback [22]. However, the fragility of the cell wall in the humanized yeast cell is still the bottleneck of this technology.

Cell wall biogenesis in yeast is regulated by the cell wall integrity signaling pathway. Rho1p is considered to be the master regulator of the cell wall integrity pathway. In response to environmental challenges, the Rho1p GTPase mobilizes and coordinates physiological actions through a variety of outputs to maintain cell wall integrity [23], however, the effect of Rho1p in glyco-engineered yeast has not yet been discussed in detail. This manuscript describes the strategy of *RHO1* overexpressing to regulate the cell wall integrity pathway in Δ*alg3*Δ*och1* double mutant *S. cerevisiae* cells, and increase the strength of cell wall to improve growth phenotype and *N*-glycosylation site occupancy.

## Results

### Overexpression of *RHO1* partially recovers the growth defect of a glycosylation mutant strain

#### Cell growth is enhanced by overexpression of RHO1

**Fig. 1:**
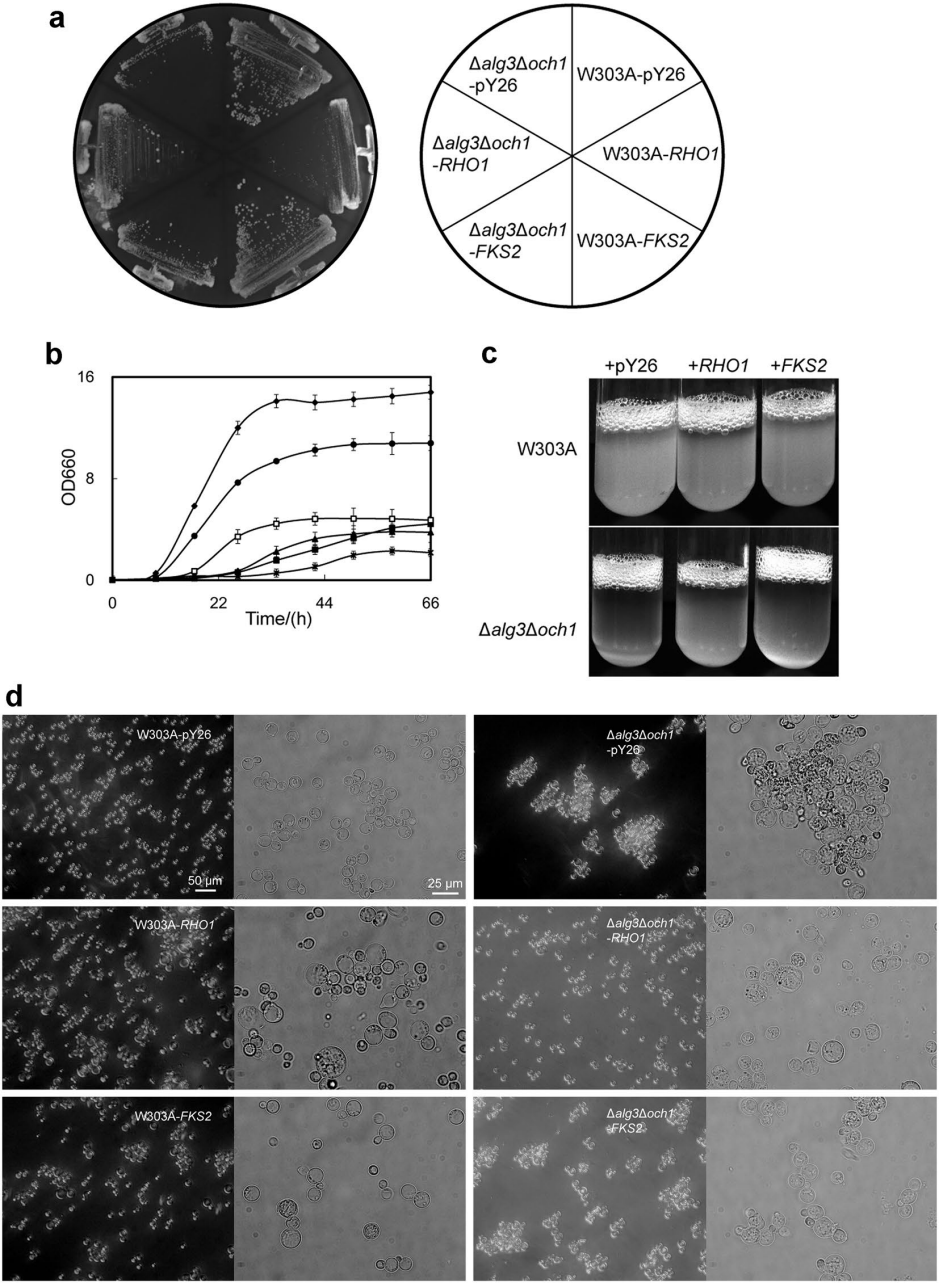
Growth phenotype of *S. cerevisiae* Δ*alg3*Δ*och1* was improved by *RHO1* overexpression. **a** *S. cerevisiae* mutant strains were prespread on SD-U (lacking uracil) solid medium at 30 °C for 48 h before single colonies were selected and streaked on SD-U plates for 24 h. **b** Growth curve of W303A-pY26 (*solid diamonds*), W303A-*FKS2* (*solid round*), W303A-*RHO1* (*snowflake*), Δ*alg3*Δ*och1*-pY26 (*solid squares*), Δ*alg3*Δ*och1*-*FKS2* (*solid triangle*), and Δ*alg3*Δ*och1*-*RHO1* (*open squares*). Cells were incubated in 5 ml of SD-U liquid medium for 24 h, and then the same amount of cells were collected and transferred into fresh 50 ml of SD-U medium for 66 h (30 °C, 230 rpm). **c** Yeast cells were cultivated in 5 ml of SD-U liquid medium for 48 h (30 °C, 230 rpm) and kept horizontally for 10 min before taking photos. **d** Morphology of mutant cells by microscope observation. Yeast Cells were incubated in 5 ml of SD-U liquid medium for 1 day (30 °C, 230 rpm). Cell morphology was determined by microscopy as described in the “Methods” section

In order to test the effects of overexpression of *RHO1* and *FSK2* genes, plasmids blank pY26, pY26-*RHO1* and pY26-*FKS2* were transformed in a wild type strain W303A or a glycosylation mutant Δ*alg3*Δ*och1* strain, respectively. The growth phenotype of these *S. cerevisiae* mutants were measured both in liquid and solid culture. The results were summarized in Fig. 1. The Δ*alg3*Δ*och1*-pY26 strain showed severe growth retardation (Fig. 1a, b) and increased flocculation (Fig. 1c). Overexpression of *RHO1* could partially recover cell growth deficiency and decrease cell flocculation in Δ*alg3*Δ*och1*, although it caused severe cell growth deficiency in wild-type W303A. The results showed that Δ*alg3*Δ*och1*-*RHO1* were grown to logarithmic phase at 17 h comparing to that of W303A-pY26 at 9 h, while Δ*alg3*Δ*och1*-pY26 exhibited a prolonged lag phase of 26 h. Δ*alg3*Δ*och1*-*RHO1* and W303A-pY26 entered stationary phase at 34 h, compared to Δ*alg3*Δ*och1*-pY26 which entered the stationary phase at 58 h. The maximum OD 660 of W303A-pY26, Δ*alg3*Δ*och1*-*RHO1* and Δ*alg3*Δ*och1*-pY26 were 14.8 ± 0.6, 4.7 ± 0.3 and 4.4 ± 0.2, respectively. These results suggested that the overexpression of *RHO1* could enhance the growth performance of Δ*alg3*Δ*och1*-pY26, although no significant improvement was observed in stationary phase cell number. However, W303A-*RHO1* showed severe growth defects, which might cause by morphological abnormalities when *RHO1* was overexpressed in normal cells. Besides, *FKS2* expressed in Δ*alg3*Δ*och1* also prevailed over the growth phenotype, though the results were not as good as *RHO1*.

#### The *och*1Δ*alg3*Δ-*RHO*1 strain is dispersed in liquid media

It has been previously reported that the *S. cerevisiae OCH1* gene is a key enzyme involved in outer chain elongation, and its disruption leads to temperature sensitivity, drug sensitivity, growth retardation, cell wall defects and cell aggregation [24, 25]. Our results were in agreement with the above reports, the *och1*Δ*alg3*Δ-pY26 double mutant strain exhibited serious cell flocculation (Fig. 1d). However, *och1*Δ*alg3*Δ-*RHO1* and -*FKS2* strains could be dispersed in the culture broth. In addition, yeast cells carrying plasmids of pY26-*RHO1* or pY26-*FKS2* were enlarged and more round than W303A-pY26 (Fig. 1d).

### Effects of *RHO1* overexpression on cell wall structure

#### RHO1 overexpression improves both the glycan and mannoprotein layer thickness

**Fig. 2:**
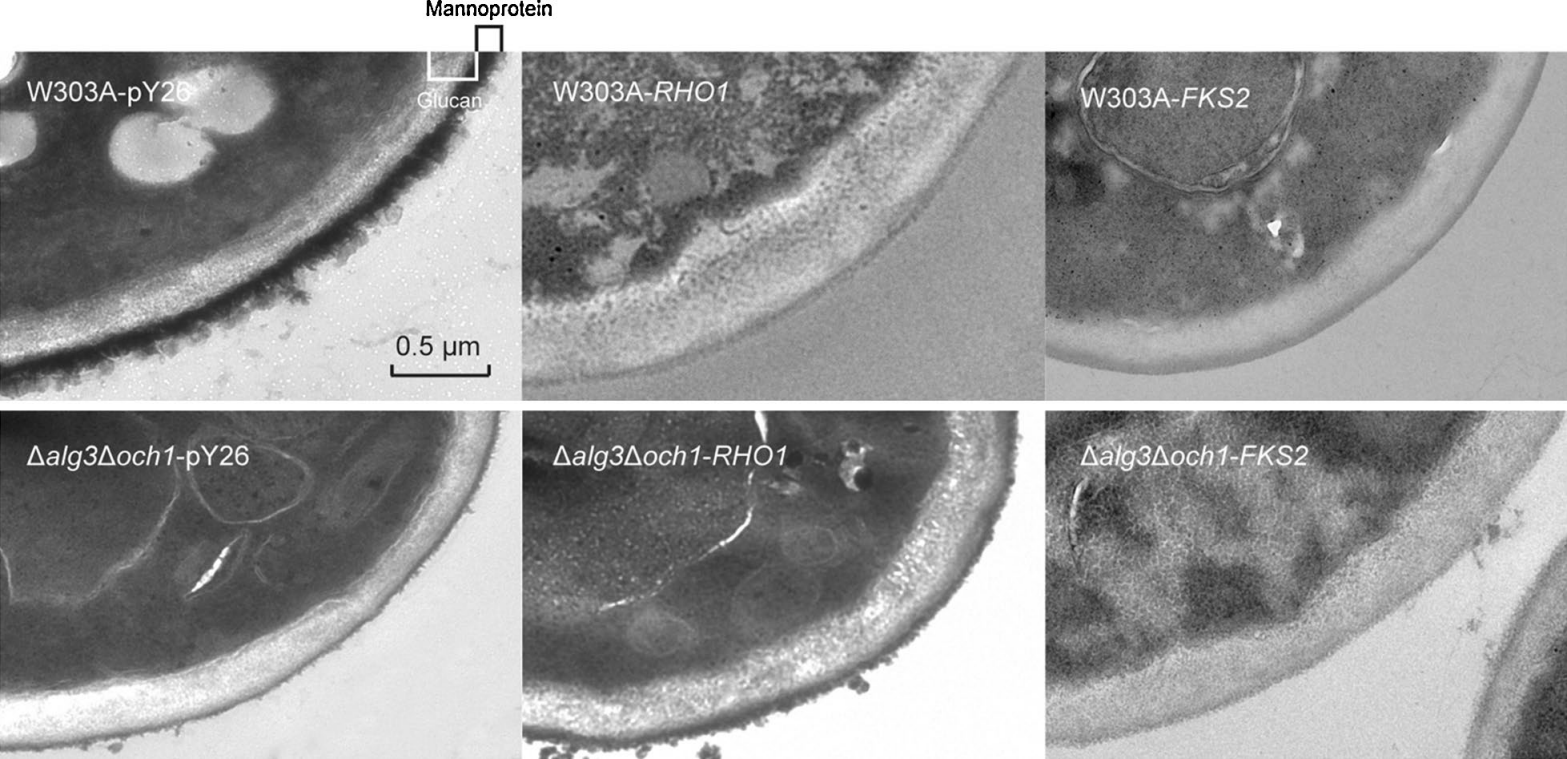
Cell wall structure were optimized by *RHO1* overexpression. TEM photographs of *S. cerevisiae* mutant strains. Cells were cultured in 5 ml of SD-U liquid medium for 1 day (30 °C, 230 rpm). Cell samples were fixed in glutaraldehyde solution as described in the “Methods” section

To confirm whether *RHO1* or *FKS2* overexpression affects cell wall structure, the cell wall of *S. cerevisiae* strains were directly observed by TEM analysis. Micrographs were taken and the thicknesses of the cell walls as well as those of the mannoprotein and glucan layers were shown in Fig. 2. Cell wall analysis of Δ*alg3*Δ*och1*-pY26 demonstrated an increase in glucan layer thickness in comparison with W303A-pY26, although the mannoprotein layer was much thinner due to the deletion of *OCH1*, which initiates heavy mannose glycosylation in yeasts. In the micrograph of *RHO1* or *FKS2* overexpression strains, an even thicker glucan layer is visible compared with W303A-pY26 and Δ*alg3*Δ*och1*-pY26, respectively. Surprisingly, an increase in mannoprotein layer thickness is also evident in the Rho1p overexpression strain compared to Δ*alg3*Δ*och1*-pY26, which might be influenced by protein glycosylation as well. In conclusion, TEM results suggest that the cell wall components could be rearranged by endogenous overexpression of Rho1p.

#### Enhanced glycan layer partially recovers the growth defect

**Fig. 3:**
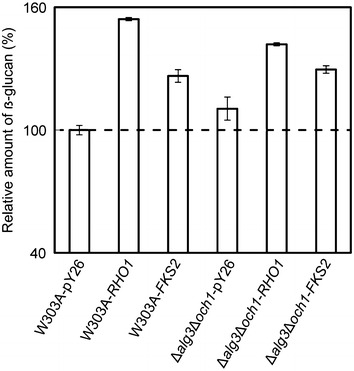
The amount of β-glucan was increased by *RHO1* overexpression. Relative β-glucan content in *S. cerevisiae*. 5 ml of yeast cells were incubated in SD-U medium for 48 h, and then transferred into fresh SD-U medium for 24 h (30 °C, 230 rpm). 2.5 × 10^6^ cells were harvested and quantity of β-glucan was determined as described in the “Methods” section

To explore the function of Rho1p and Fks2p on β-glucan accumulation, the amount of β-glucan in these mutant cells were measured. The relative content of β-glucan was defined to be 100 ± 2.2 % in the cell wall of W303A-pY26, thus Δ*alg3*Δ*och1*-pY26 was calculated to contain 110.5 ± 5.7 % β-glucan (Fig. 3). Overexpression of *RHO1* in W303A and Δ*alg3*Δ*och1* increased the relative β-glucan content by 54.2 ± 0.7 and 41.9 ± 0.7 %, respectively. These results well corresponded to that of the above mentioned microscopic results. Rho1p is an essential component of the 1,3-β-glucan synthase complex (GS complex) [26], thus it was not surprising that *RHO1* overexpression could enhanced the glucan content of yeast cells. Meanwhile, the *FKS2* gene which encodes another component of GS complex was also overexpressed in this study, the results showed that the β-glucan content was increased by 26.5 ± 3.1 % in W303A-*FKS2* and 29.6 ± 1.8 % in Δ*alg3*Δ*och1*-*FKS2*, respectively, suggesting that the thickening of the glucan layer was due to the activity of yeast GS complex.

To examine the extent of *RHO1* and *FKS2* expression in recombinant strains, the relative expression changes of *RHO1* and *FKS2* were determined by quantitative real-time RT-PCR. As shown in Table 1, *RHO1* in W303A-*RHO1* and Δ*alg3*Δ*och1*-*RHO1* strains were up-regulated 72.0 and 6.4-fold differential expression respectively, comparing with W303A-pY26, and the observed significant increase in expression can be attributed to *RHO1* overexpression. In addition, a moderate increase of *FKS2* expression was also observed in *FKS2*-overexpression strains. However, it should be noted that the *och1* and *alg3* double mutant also lead to moderate increase in transcriptional levels of *RHO1* and *FKS2* genes.

**Table 1:** Differential expression of *RHO1* and *FKS2* in *S. cerevisiae* mutant strains

Strains^a^	Relative expression levels	
	*RHO1*	*FKS2*
W303A-pY26	1.00	1.00
W303A-*RHO1*	72.00	2.35
W303A-*FKS2*	−1.16	5.28
Δ*alg3*Δ*och1*-pY26	3.20	1.56
Δ*alg3*Δ*och1*-*RHO1*	6.45	2.36
Δ*alg3*Δ*och1*-*FKS2*	3.39	2.66

^a^Yeast cells were pre-cultured for 48 h and transferred to fresh SD-U medium for 24 h (30 °C, 230 rpm)

### Effects of *RHO1* overexpression on cell wall integrity

**Fig. 4:**
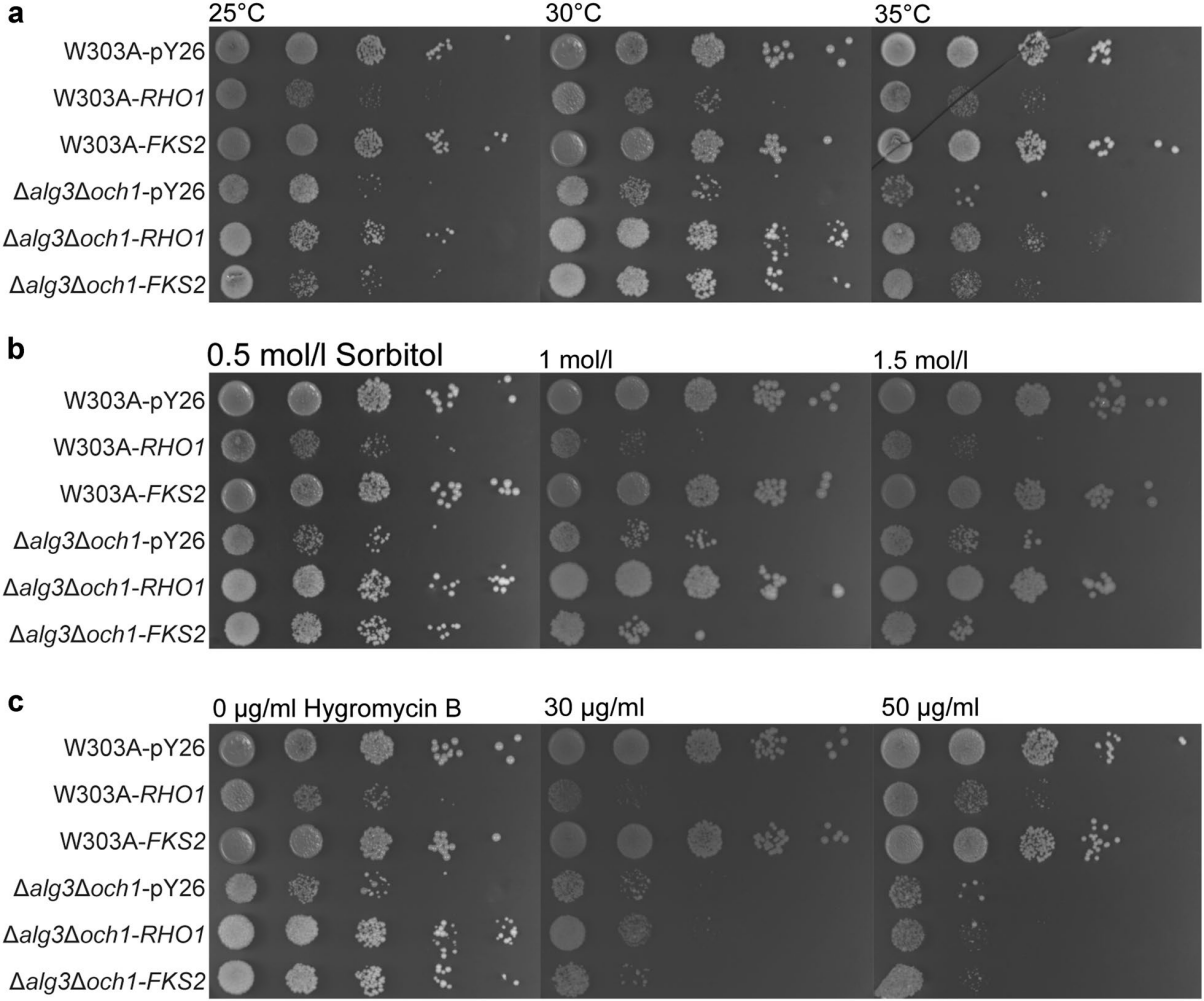
Assay results for sensitivities to different stress. **a**–**c** Resistance of W303A-pY26, W303A-*RHO1*, W303A-*FKS2*, Δ*alg3*Δ*och1*-pY26, Δ*alg3*Δ*och1*-*RHO1 *and Δ*alg3*Δ*och1*-*FKS2* to different temperature, sorbitol content and hygromycin B content. Cells were pre-incubated in SD-U liquid medium for 1 day (30 °C, 230 rpm). Serial dilutions (1/10 each time) of each culture were spotted onto SD-U plates and incubated with different stress reagents or temperature for another 48 h

To analyse the phenotypes caused by Rho1p or Fks2p expressing, a series of *RHO1* or *FKS2* overexpression strains were subjected to multiple environmental stresses, such as temperature tolerance, osmotic stress and hygromycin B sensitivity (Fig. 4a–c). The *RHO1*-overexpressing cells exhibited a significant growth recovery when subjected to the above environmental stress, compared to Δ*alg3*Δ*och1*-pY26. However, Δ*alg3*Δ*och1*-*FKS2* showed more stress sensitivity than Δ*alg3*Δ*och1*-*RHO1*. Thus it can be supposed from our results that as cell wall integrity signaling is induced in response to a variety of cell wall stresses, the overexpression of the gene that encodes Rho1p GTPase which triggers the cell wall integrity pathway might mobilize and coordinate physiological actions to resist environmental challenges.

### Effects of *RHO1* overexpression on production of glycoprotein

#### Improvement of *N*-glycan site occupancy by Rho1p overproduction

**Fig. 5:**
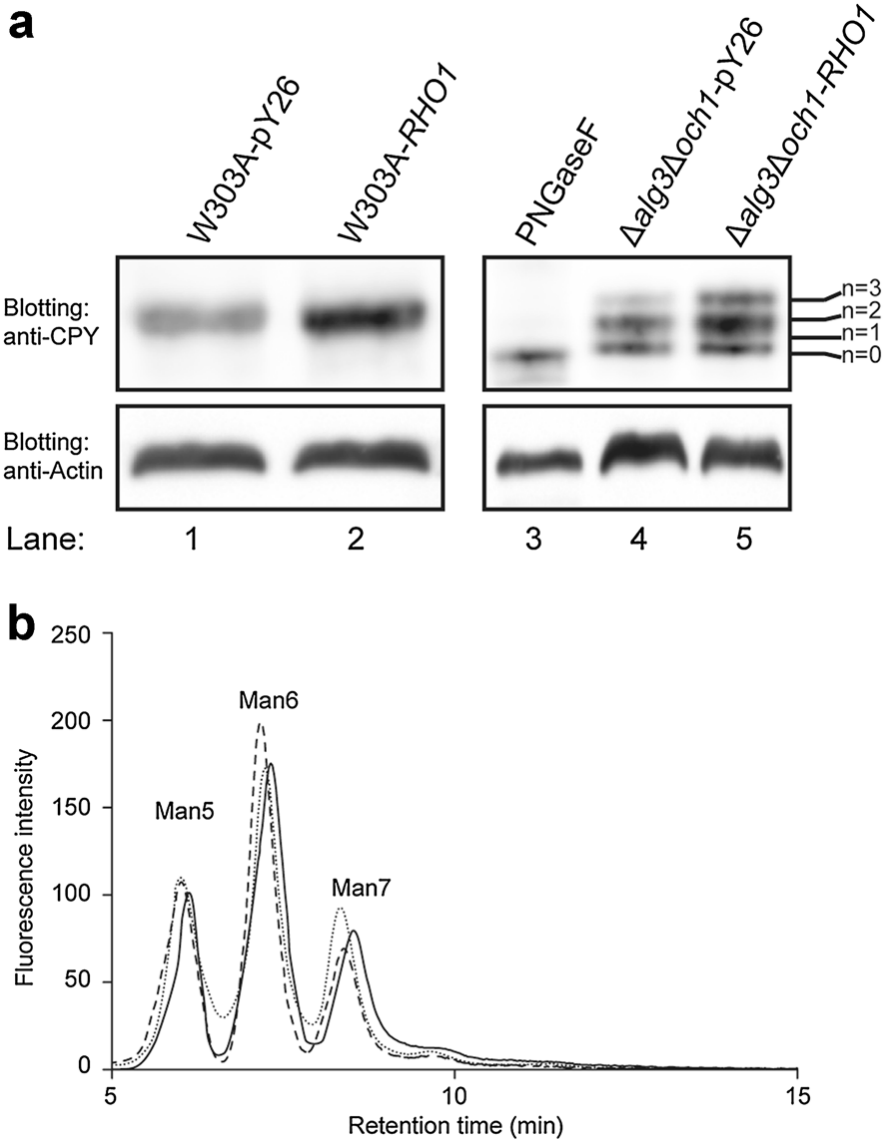
Comparative analysis of glycosylation occupancy and glycan patterns of W303A-pY26, W303A-*RHO1*, Δ*alg3*Δ*och1*-pY26 and Δ*alg3*Δ*och1*-*RHO1*.** a** Analysis of glycosylation occupancy of CPY proteins. 5 ml of yeast cells were incubated in SD-U medium for 48 h, and then transferred into fresh SD-U medium for another 24 h (30 °C, 230 rpm). 5 ml of growing cells were harvested and washed twice with deionized water. Intracellular proteins were extracted and analyzed by western blotting with anti-ScCPY antibody as described in the “Methods” section. The number of *N*-glycosylation sites is indicated by ‘n’. **b** Analysis of *N*-glycan profiles by HPLC. Δ*alg3*Δ*och1*-pY26 (*dotted line*), Δ*alg3*Δ*och1*-*RHO1* (*line segment*), and Δ*alg3*Δ*och1* (*solid line*) were cultured in 5 ml of SD-U liquid medium at 30 °C for 48 h, and then transferred to fresh 50 ml of SD-U medium for 24 h. *N*-glycans were extracted from 5 ml of each culture. Three peaks represent Man_5_GlcNAc_2_, Man_6_GlcNAc_2_ and Man_7_GlcNAc_2_, respectively

Disruption of the *ALG3* gene results in the modification of proteins mainly with Man_5_GlcNAc_2_, which causes under-occupancy of glycosylation sites [27]. However, an increase in mannoprotein layer thickness in Δ*alg3*Δ*och1*-*RHO1* was detected by TEM analysis (Fig. 2). To analyze whether *RHO1* overexpression affected the glycosylation levels, western blotting assays were performed. The mobility pattern of carboxypeptidase Y (CPY) carrying four *N*-glycosylation sites was analysed in this work as a model glycoprotein. In W303A-pY26 and W303A-*RHO1*, CPY was full glycosylated, thus only one band was shown by western blotting, respectively. Compared to Δ*alg3*Δ*och1*-pY26, Δ*alg3*Δ*och1*-*RHO1* strain showed increased *N*-glycan occupancy of CPY. One to two *N*-glycosylation sites were detected in Δ*alg3*Δ*och1*-pY26 (Fig. 5a), indicating low glycosylation efficiency, which normally occurs in glycosylation deficient mutants [28]. However, in the case of the Rho1p overproduction strain, it apparently generates one to three occupancy sites of CPY at *N*-glycosylation sites (Additional file 1: Figure S1).

Furthermore, the structure of the *N*-glycans in cell wall mannoprotein were analyzed, using a HPLC-based method. The oligosaccharides from Δ*alg3*Δ*och1* and Δ*alg3*Δ*och1*-pY26 revealed several peaks corresponding to Man_5_GlcNAc_2_, Man_6_GlcNAc_2_ and Man_7_GlcNAc_2_. Moreover, the oligosaccharides from Δ*alg3*Δ*och1*-*RHO1* did not reveal a glycoform that was different from the above two strains, but merely exhibited enhanced amounts of glycans (Fig. 5a, b). These results suggest that *RHO1* overexpression affects the efficiency of glycosylation, which is mainly due to the attachment of shorter oligosaccharides but has no impact on the pattern of the glycan chains.

#### RHO1 overexpression altered protein secretion

**Fig. 6:**
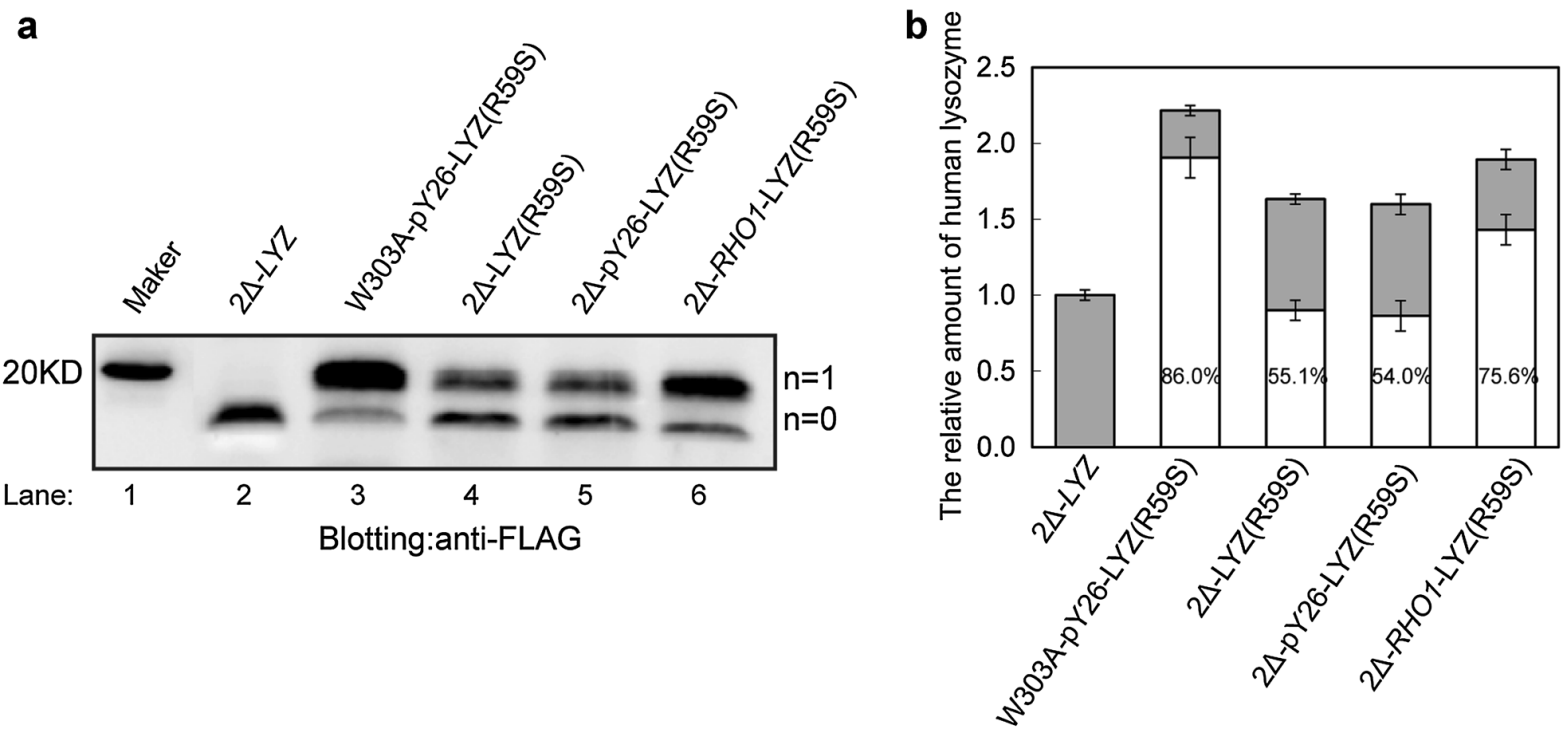
Secreted and *N*-glycosylated human lysozyme was increased by *RHO1* overexpression. **a** Analysis of human lysozyme secretion by western blotting. 5 ml of cells were cultured in SD-UT (lacking uracil and tryptophan) liquid medium for 48 h and transferred into 50 mL fresh SD-UT (30 °C, 230 rpm) for 24 h. The secreted human lysozyme was collected from 30 ml of each supernatant and concentrated as described in the “Methods” section. The concentrated supernatant was analyzed by Western blotting with anti-FLAG antibody. 2Δ: Δ*alg3*Δ*och1*. **b** Analysis of the relative content of human lysozyme. The ratios of human lysozyme were calculated by ImageQuant™ Software version 7.0. The relative content of lysozyme in the lane of Δ*alg3*Δ*och1*-LYZ was defined to be 1 ± 0.03. *White* glycosylated human lysozyme; *Grey* non-glycosylated human lysozyme

The ability of the Δ*alg3*Δ*och1*-*RHO1* strain to secrete human lysozyme (hLYZ) as a measurement of glycoprotein product was analyzed thereafter. Wild type human lysozyme is a non-glycosylated protein, in this work one *N*-glycosylation site was introduced by site-directed mutagenesis. The glycosylated lysozyme illustrated a higher molecular weight band, and as demonstrated by Western blot analysis (Fig. 6a), Δ*alg3*Δ*och1*-*RHO1* secreted much more glycosylated lysozyme than both Δ*alg3*Δ*och1* and Δ*alg3*Δ*och1*-pY26. In Δ*alg3*Δ*och1*-*RHO1*, 75.6 % of human lysozyme was glycosylated, compared to 55.1 % in Δ*alg3*Δ*och1* and 54.0 % in Δ*alg3*Δ*och1*-pY26 (Fig. 6b). The ratios of lysozyme in Δ*alg3*Δ*och1*, Δ*alg3*Δ*och1*-pY26 and Δ*alg3*Δ*och1*-*RHO1* were 1:1:1.2, suggesting Rho1p positively influenced secretion of foreign glycoproteins. Based on these results, along with the glycosylation occupancy experiments and cell wall structure micrographs, we can be concluded that the overexpression of *RHO1* in *och1*Δ*alg3*Δ, not only could improve the cell growth, but also could enhance the *N*-glycan occupancy and the secretion ability of glycoproteins.

## Discussion

With the increasing importance for production of humanized glycans for therapeutic purposes, constructing a suitable expression system is necessary. Upon disruption of *OCH1*, a loss of hyper-mannosylated structure in secreted glycoproteins was observed [29], leading to a defective mannoprotein layer in the Δ*alg3*Δ*och1* cell wall. The fungal cell wall, which comprises 20–30 % of cell dry weight, is mainly composed of 1,3-β-glucan, 1,6-β-glucan, mannoproteins and chitin [18]. In this study, overexpression of *RHO1* has been proposed as a novel strategy to compensate for mannoprotein layer defects. Rho1p regulates both 1,3-β-glucan synthase encoded by the *FKS1* and *FKS2* genes and the 1,6-β-glucan synthase [23]. As a result, overexpression of *RHO1* in Δ*alg3*Δ*och1* improved the amount of glucan and the glucan layer thickness of the cell wall, which partially recovered the cell wall defect. Similar phenomenon have been described previously in *S. pombe*, the cell wall tends to be thicker than wild-type cells when *RHO1* is overexpressed [30]. Interestingly, cell aggregation of glycosylation defective cells was also alleviated by the direct function of Rho1p in the cell wall, suggesting that an enhancement of glucan layer could partially strengthen the yeast cell wall.

As an Δ*alg3*Δ*och1*-*RHO1* strain is planned to be further reconstructed and engineered for industrial use, the cell growth rate is considered to be an extremely important parameter for further application. *RHO1* overexpression enhanced the growth phenotype of Δ*alg3*Δ*och1* by both reducing the time course of log phase and improving the cell growth rate. In addition, when subjected to thermal, osmotic or drug stress, the growth phenotype was also improved by *RHO1* overexpression. Contrarily, by overexpression of *FKS2*, which encodes a catalytic subunit of a 1,3-β-glucan synthase, no significant growth enhancement was detected upon extreme environmental stress, although the cell growth of Δ*alg3*Δ*och1*-*FKS2* was improved both in liquid and solid media under normal condition. As both *RHO1* and *FKS2* overexpression led to glucan accumulation in the cell wall, the reason why the stress resistance of Δ*alg3*Δ*och1*-*RHO1* was increased could not be simply explained by the partial recovery of the cell wall. Studies have shown that Rho1p is an essential protein which controls cell wall integrity signaling and linear series of protein kinases, known as the MAP kinase cascade, is responsible for amplification of the cell wall integrity signal from Rho1p [31]. When yeast cells with weakened cell walls suffer external stress, they commonly activate the cell wall integrity pathway [18] which was strengthened by *RHO1* overexpression, thus leading to an obvious stress resistance enhancement of Δ*alg3*Δ*och1*-*RHO1*. For industrial strains, easily suffered with a variety of different stresses from culture media, the stress-resistance ability in Δ*alg3*Δ*och1*-*RHO1* would be a considerable advantage in its industrial use.

Upon disruption of the *ALG3* gene in yeasts, the reduced occupancy of *N*-glycosylation sites was observed [12]. However, consistent and robust *N*-glycan site occupancy is desirable for the production of therapeutic glycoproteins in order to maintain desired product profiles. *N*-glycan site occupancy of glycoproteins is affected by the dolichol biosynthetic pathway, oligosaccharyltransferase complex, the target polypeptide sequence, and structure of oligosaccharides [10, 32]. De Pourcq et al. overexpressed *ALG6* in a *Y. lipolytica* Δ*alg3* strain to modify the target oligosaccharides structure and enhance site occupancy [27]. Here, an alternative method is provided to remedy under-occupancy in CPY by overexpression of the *RHO1* gene. Based on previous reports that oligosaccharyltransferase may be subject to regulation by the cell wall integrity pathway via an interaction between Pkc1p with Stt3p [18] and Rho1p interacts with Pkc1p by two-hybrid analyses in *S. cerevisiae* [33], it is predicted that Rho1p might indirectly regulate the activity of oligosaccharyltransferase to induce the *N*-glycan occupancy of glycoproteins. Besides, Rho1p regulates the 1,6-β-glucan synthase which has not yet been described at the molecular level [23], and 1,6-β-glucan possesses the function to anchor mannoproteins to other cell wall components [34]. It was considered that *RHO1* overexpression might activate the production of 1,6-β-glucan, and increase mannoprotein attachment as a result. This speculation might be verified by the cell wall morphology of Δ*alg3*Δ*och1*-*RHO1* cells which were partially restored to that of W303a-pY26 with an increasing thickness in mannoprotein layer when compared to Δ*alg3*Δ*och1*.

In addition, *S. cerevisiae* Δ*alg3*Δ*och1* with *RHO1* overexpression secreted more exogenous proteins. Rho1p has been proposed to be responsible for the spatial regulation of the exocyst complex [31]. The exocyst mediates polarized targeting and tethering of post-Golgi secretory vesicles to sites of exocytosis prior to SNARE-mediated fusion [35]. Thus, it is possible that the exocyst complex was activated by *RHO1* overexpression to mediate more secreted glycoproteins to the cell surface. The results presented here provide a comprehensive and convenient strategy to combine improved cell growth, stress resistance, *N*-glycan site occupancy and secretion of foreign proteins in glycosylation defect strains. The authors believe that it may significantly facilitate its industrial applications and could be extended to other yeast expression systems as well.

## Conclusions

In summary, *RHO1* was overexpressed in Δ*alg3*Δ*och1* in this study to improve the amount of cell wall content of glucan, thus further enhanced the cell growth phenotype and stress resistance. Moreover, overexpression of *RHO1* increased *N*-glycan site occupancy on vacuolar carboxypeptidase Y protein, as judged by its mobility pattern and also the amount of secreted glycoproteins in the medium. To the best of our knowledge, this study reports for the first time to enhance the cell growth, *N*-glycan occupancy and secretion ability simultaneously by simply overexpressing of *RHO1* in *S. cerevisiae*.

## Methods

### Plasmids, strains and culturing conditions

The plasmids and yeast strains used in this study are described in Table 2. Standard genetic techniques were used unless otherwise noted [36]. *Saccharomyces cerevisiae* strain W303-1A was used as the host. *Saccharomyces cerevisiae* W303-1A genome DNA was used for amplifying the *RHO1* and *FKS2* sequences. pY26-*RHO1* and pY26-*FKS2* were used to express *RHO1* or *FKS2* under control of the *GPD1* promoter, respectively. pY26-*RHO1*, pY26-*FKS2* and blank pY26 [37] were transformed into Δ*alg3*Δ*och1* strain, designated as Δ*alg3*Δ*och1*-*RHO1*, Δ*alg3*Δ*och1*-F*KS2* and Δ*alg3*Δ*och1*-pY26, respectively.

**Table 2:** Plasmids and *S. cerevisiae* strains used in this study

	Description	Source
*Plasmid*		
pY26TEF-GPD (pY26)	*URA3*/2μ yeast shuttle vector containing *GPD1* promoter	[37]
pY26-*RHO1*	*RHO1* expressed from *GPD1* promoter in pY26TEF-GPD	This study
pY26-*FKS2*	*FKS2* expressed from *GPD1* promoter in pY26TEF-GPD	This study
pRS424TEF	*TRP1*/2μ yeast shuttle vector containing *TEF1* promoter	[38]
pRS424TEF-LYZ	hLYZ expressed from *TEF1* promoter in pRS424TEF	This study
pRS424TEF-LYZ(R59S)	hLYZ(R59S) expressed from *TEF1* promoter in pRS424TEF	This study
*Strains*		
W303A-pY26	As in W303-1A and blank pY26TEF-GPD	This study
W303A-*RHO1*	As in W303-1A and pY26-*RHO1*	This study
W303A-*FKS2*	As in W303-1A and pY26-*FKS2*	This study
Δ*alg3*Δ*och1*-*RHO1*	As in Δ*alg3*Δ*och1* and pY26-*RHO1*	This study
Δ*alg3*Δ*och1*-*FKS2*	As in Δ*alg3*Δ*och1* and pY26-*FKS2*	This study
Δ*alg3*Δ*och1*-pY26	As in Δ*alg3*Δ*och1* and blank pY26TEF-GPD	This study
Δ*alg3*Δ*och1*-LYZ	As in Δ*alg3*Δ*och1* and pRS424TEF-LYZ	This study
W303A-pY26-LYZ(R59S)	As in W303A with blank pY26TEF-GPD and pRS424TEF-LYZ (R59S)	This study
Δ*alg3*Δ*och1*-LYZ(R59S)	As in Δ*alg3*Δ*och1* and pRS424TEF-LYZ (R59S)	This study
Δ*alg3*Δ*och1*-pY26-LYZ (R59S)	As in Δ*alg3*Δ*och1* with pRS424TEF-LYZ (R59S) and blank pY26TEF-GPD	This study
Δ*alg3*Δ*och1*-*RHO1*-LYZ (R59S)	As in Δ*alg3*Δ*och1* with pRS424TEF-LYZ (R59S) and pY26-*RHO1*	This study

pRS424TEF-LYZ(R59S) [38] was used to express a mutated human lysosome (hLYZ), in which 59th Arg of hLYZ is mutated to Ser, under the control of *TEF1* promoter. To construct this, first, site-directed mutagenesis on hLYZ was performed by using QuikChange™ site-directed mutagenesis kit (Stratagene, La Jolla, California). The Asn_57_-Thr_58_-Arg_59_ polypeptide segment was mutated into Asn_57_-Thr_58_-Ser_59_ and Asn_57_ (57th amino acid of human lysosome) was able to be *N*-glycosylated. All plasmids constructed in this experiment have been verified by Sanger sequencing (Sangon, Shanghai, China). Plasmid maps and primer sequences are available on request.

Synthetic dropout medium (SD, 0.67 % yeast nitrogen base, 2 % glucose) media with appropriate supplemental amino acids were used to culture yeast cells and to select yeast transformants.

### Real-time quantitative (RT-PCR)

Total RNA was extracted from a series of engineered *S. cerevisiae* mutant strains using GenElute™ mRNA Miniprep Kit (Sigma-Aldrich, USA) according to the manufacturer’s protocol. The yield of RNA was determined using a NanoDrop 2000 spectrophotometer (Thermo Scientific, USA), and the integrity was evaluated using agarose gel electrophoresis stained with ethidium bromide. Complementary DNA (cDNA) was generated by PrimerScript RT Enzyme Mix I (TaKaRa, Japan). 0.5 μg RNA was used as template and oligo (dT) was used as a primer. The cDNA was then diluted ten times in nuclease-free water and stored at −20 °C.

Real-time PCR was performed using LightCycler^®^ 480 Real-time PCR Instrument (Roche, Switserland); 2 × LightCycler^®^ 480 SYBR Green I Master (Roche, Swiss) was used as a PCR reagent. The primer sequences listed in Table 3 were designed in the laboratory and synthesized by Generay Biotech (Generay, PRC) based on the mRNA sequences obtained from the NCBI database. The expression levels of mRNAs were normalized to actin gene *ACT1* and were calculated using the 2^−ΔΔCt^ method [39].

**Table 3:** Primers and amplification product sizes for each gene in quantitative real-time RT-qPCR analysis

Gene	Forward primer	Reverse primer	Product length(bp)
*RHO1*	GAATGTTCGGCCAAGACTG	CTTCTTAGCTTTACCATTCGTT	102
*FKS2*	GGTGGTCGTATCAAGCAT	CCATACCAGCACCGATCTTA	103

### Measurement of yeast growth

The growth rate of yeast cells were determined by measuring OD_660_ after appropriate dilutions using a spectrophotometer (Ultrospec 2100 Pro, GE healthcare, Fairfield, USA) every 8 h. The measurements were continued until the OD660 nm reached the plateau. This experiment was repeated 3 times. The growth curve of different strains was drawn by software of Excel Microsoft Office Professional Plus 2013 (Microsoft, Redmond, USA).

### Extraction of intracellular carboxypeptidase Y

The cells were collected by centrifugation (10,000×*g*, 1 min) and then incubated in 1 ml lyticase buffer (1.0 M sorbitol, 2 mM MgCl_2_, 0.14 % β-mercaptoethanol, 50 mM Tris–HCl, pH 7.5) with lyticase (Sigma-Aldrich, Shanghai, China) at a concentration of 50 U/OD_600_. After incubation (30 °C, 60 min) most of the cells were converted to spheroplasts. The spheroplasts were collected and washed twice with ice-cold lysis buffer (0.2 M sorbitol, 1 mM EDTA, 50 mM Tris–HCl, pH 7.5). Spheroplasts were then suspended in 500 μl lysis buffer containing protease inhibitors (1 mM PMSF) and an appropriate amount of glass beads. The mixture was incubated on ice for 30 s and vortexed 30 s for about five to seven cycles (URBomix VORTEX-GENIE, Scientific Industries, USA). Cells were lysed and centrifuged (1000×*g,* 4 °C) for 10 min to remove unlysed cells and cell wall debris. The suspensions were carefully collected as whole intracellular protein. Protein concentration was determined by a bicinchoninic acid (BCA) protein assay kit (Beyotime, Jiangsu, China). About 10 μg of protein was separated via SDS-PAGE for western blotting with anti-CPY antibody (Thermo Scientific, Waltham, USA) as described below.

### Detection of secreted human lysozyme

The culture media was collected by centrifugation (1000×*g*, 10 min) and the cell pellet was removed. The spent media was then centrifuged (4000×*g*, 4 °C) with an Amicon-Ultra Centrifugal Filter (molecular weight cut off 10 kD, Millipore, Shanghai, China) and concentrated into 250 μl. Protein concentrations were determined as above. About 10 μg of protein was separated via SDS-PAGE and human lysozyme was detected by western blotting with anti-FLAG antibody (Transgen, Beijing, China) as described below. This experiment was repeated 3 times.

### Western blot analysis

Proteins were suspended in sample buffer (2 % SDS, 5 % glycerol, 5 % 2-mercaptoethanol, 0.002 % bromophenol blue, 62.5 mM Tris–HCl, pH 6.8) and incubated in 100 °C for 3 min. Proteins were separated in 10 or 12 % SDS–polyacrylamide gels and blotted onto PVDF membranes. For blocking Tris-buffered saline containing 0.5 % Tween 20 (TBST) and 5 % dry milk (Trans-Blot Turbo system, Biorad, USA) was used. The appropriate primary antibodies were diluted in TBST and 5 % dry milk followed by incubation with secondary antibodies. Signals were visualized by Clarity Western ECL Substrate (BioRad, Shanghai, China) and images were obtained using ImageQuant LAS 4000 mini (GE Healthcare Bio-Science, Stockholm, Sweden).

### Quantitative measurements of β-glucan

The amount of β-glucan per gram cells were measured using aniline blue as described previously with some modifications [40, 41]. Cells were grown to early log phase (2.5 × 10^6^ cells) and harvested (10,000×*g*, 1 min). The cells were washed twice with 1 ml TE buffer (10 mM Tris–HCl, 1 mM EDTA, pH = 8.0), resuspended to 250 μl in TE and then 6 M NaOH was added to a final concentration of 1 M. Following incubation in a water bath at 80 °C for 30 min, 1.05 ml of AB mix [0.03 % aniline blue (Sigma-Aldrich, USA), 0.18 M HCl, and 0.49 M glycine/NaOH, pH 9.5] was added. The tube was vortexed briefly and then incubated at 50 °C for 30 min. Fluorescence of β-glucan was quantified using a spectrofluorophotometer (Ultrospec 2100 Pro, GE healthcare, Fairfield, USA). Excitation wavelength was 400 nm and emission wavelength was 460 nm. This experiment was repeated three times.

### Transmission electron microscopy and light microscopy

Transmission electron microscopy (TEM) analysis of the *S. cerevisiae* cell wall was carried out as described by Bzducha-Wrobel et al. [42]. For microscopic observations, yeast cells in glutaraldehyde prepared in phosphate buffer (pH 7.2) were fixed in osmium solution (OsO_4_). After rinse with cold water and dehydration with ethanol, the samples were embedded in Epon resin. Ultrathin sections were prepared by means of an ultramicrotome (Leica UC6, Biberach, Germany). Thereafter, the sections were stained as described by Deryabina et al. [43] and examined by TEM (Hitachi HT7700, Ibaraki, Japan).

Light microscopic images were obtained using a Nikon Eclipse Ti-E inverted microscope equipped with DS-Ri1 camera and NIS-Element AR software (Nikon, Tokyo, Japan).

### HPLC analysis of Asn-linked oligosaccharides on mannoproteins

Cells were harvested (10,000×*g*, 1 min) and washed with deionized water three times and then centrifuged (5000×*g*, 4 °C, 10 min). Cells treated with 4 mL of 100 mM citrate buffer (pH 7.0) per 1 g pellet, were then autoclaved (121 °C, 120 min). The supernatant was recovered by centrifugation. Three volumes of cold ethanol were added to the supernatant, which was then kept on ice for 5 min. The pellet was recovered by centrifugation, dissolved again in water and freeze-dried (EYELA FD-1000 freeze dryer, Tokyo Rikakikai, Tokyo, Japan). To collect glycans, the pellet was treated with glycopeptidase F (Takara, Dalian, China) and pyridylamination was carried out using a commercially available reagent kit (Takara, Dalian, China). Pyridylaminated oligosaccharides were analyzed by HPLC using a size-fractionation column (TSKgel Amide-80, TOSOH, Japan) [44].

## Additional file

**Additional file 1: Figure S1.** Comparative analysis of glycosylation occupancy of Δ*alg3*Δ*och1*, Δ*alg3*Δ*och1*-pY26 and Δ*alg3*Δ*och1*-*RHO1*.

## Abbreviations

CHO: Chinese hamster ovary
ER: endoplasmic reticulum
GS complex: 1,3-β-glucan synthase complex
CPY: carboxypeptidase Y
hLYZ: human lysozyme
SD: synthetic dropout medium
RT-PCR: real-time quantitative
cDNA: complementary DNA
BCA: bicinchoninic acid
TEM: transmission electron microscopy
