# Supplementary figures and images for: Effects of Rho1, a small GTPase on the production of recombinant glycoproteins in Saccharomyces cerevisiae
Source: Microb Cell Fact. 2016 Oct 21;15:179. doi: 10.1186/s12934-016-0575-7 (PMC5073930; doi:10.1186/s12934-016-0575-7)

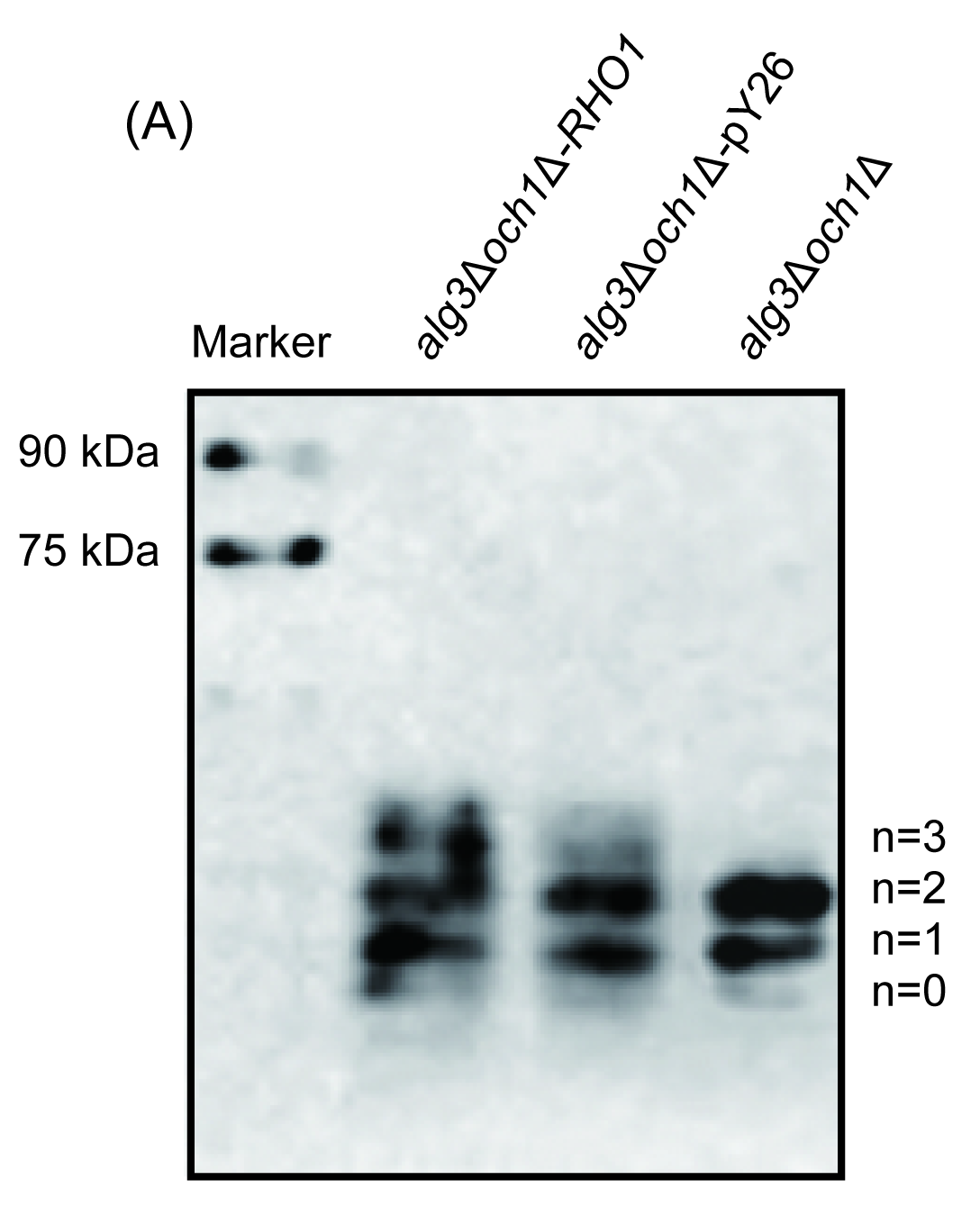

Supplement: Supplementary file 1 — Additional file 1: Figure S1. Comparative analysis of glycosylation occupancy of Δalg3Δoch1, Δalg3Δoch1-pY26 and Δalg3Δoch1-RHO1. [file 12934_2016_575_MOESM1_ESM.tif]
